# Posterior vitreous detachment and retinal tear – a prospective study of community referrals

**DOI:** 10.1038/s41433-023-02779-3

**Published:** 2023-10-05

**Authors:** Thomas R. W. Nixon, Rebecca L. Davie, Martin P. Snead

**Affiliations:** 1 Vitreoretinal Research Group, John van Geest Centre for Brain Repair, University of Cambridge, Forvie Site, Robinson Way, Cambridge, CB2 0PY UK; 2 School of Clinical Medicine, University of Cambridge, Addenbrooke’s Hospital, Hills Road, Cambridge, CB2 0SP UK

**Keywords:** Eye manifestations, Retinal diseases

## Abstract

**Background:**

Retinal tears (RT) from posterior vitreous detachment (PVD) are an important and treatable cause of rhegmatogenous retinal detachment (RRD). Better understanding of the risk of RT from PVD will help plan urgent eye care.

**Methods:**

Prospective observational case series over two years. Patients presenting to their optometrist, family doctor or emergency department with flashes and floaters were directed to a research clinic. History and examination, including slit-lamp biomicroscopy (SLB) and indentation indirect ophthalmoscopy (IIO), were performed by a single investigator, with two month follow-up for patients with confirmed PVD. Main outcome measures were incidence of PVD, RT, and RRD.

**Results:**

1010 patients were recruited. 896 (89%) patients had PVD at first assessment, of which 89 (8.8% of total cohort, 9.9% of PVD eyes) had RT and 8 had RRD. 21 (3%) of the remaining PVD patients developed RT in the subsequent two months and a further 9 (11%) patients with RT at initial assessment developed further tears by two months. 7 (0.7%) had asymptomatic RT in the fellow eye. 15% of RT were only visible on IIO and not SLB. Weiss ring was absent in 32% of eyes with RT. Patients with RT or RRD were more likely than ‘PVD-only’ eyes to have blurred or missing vision (*p* < 0.001), have higher rate of blue-green cataracts (*p* < 0.001), and longer axial lengths (*p* < 0.05).

**Conclusions and Relevance:**

This large, prospective study demonstrates a 9.9% rate of RT or RRD at the time of PVD, and emphasises the importance of IIO examination.

## Introduction

Symptomatic posterior vitreous detachment (PVD) (photopsia and/or increased floaters) is a common presentation to community optometric practices and primary eye care services. The diagnosis and management is important as it can be associated with retinal tears and rhegmatogenous retinal detachment (RRD) [1], a condition that is blinding without surgical repair. Untreated, new retinal tears can progress to retinal detachment in 30–47% of cases [2, 3], but with timely accurate retinopexy, the risk of RRD reduces to 2.1-8.8% [3, 4]. It is therefore critical to detect tears resulting from PVD before they progress to retinal detachment. Photopsia and floaters may also be associated with other conditions including intra-ocular inflammation, physiological changes in the vitreous gel (syneresis) and neurological conditions such as migraine [5]. Clinical diagnosis of PVD includes an enquiry about the patient’s symptoms, past ocular and family ocular history together with a clinical examination with dilated fundoscopy. Binocular indirect ophthalmoscopy with scleral indentation (IIO) is the gold-standard for examination of patients harbouring potentially sight-threatening retinal tears, as a minority of tears may not be visible using other methods, but there is little consensus on what constitutes acceptable practice in clinical settings as scleral depression combined with indirect ophthalmoscopy is a skill requiring thorough training and such expertise may not be available in a community primary eye-care setting. Ultra-widefield imaging was not used in this study and is not part of our routine clinical practice for diagnosing retinal tears. It is limited in its ability to see the retina out to the ora serrata, particularly in the vertical meridian, and can miss nearly half of RTs [6]. Indentation also has the key advantage of providing a dynamic examination, whereby the operculum or flap of occult very small retinal tears can be shown in relief.

Previous studies on acute PVD have potential limitations, sometimes being based in specialist tertiary retinal practices [7, 8], with possible referral bias with a higher incidence of pathology than may be found in all patients in the general community, and are either prospective but small [5, 9–11] or large but retrospective [12–16]. Increasing demands on resources means that opinion is divided as to the need or otherwise for patients with acute PVD to be followed up to detect delayed RT [11, 13, 14].

In order to address these important issues and plan how referral pathways and services are structured, it would be helpful to have definitive data from a large prospective study of acute PVD in a community, non-tertiary referral setting. This study addresses that need.

The primary study aim was to determine the incidence of retinal tears associated with acute PVD in a large general population cohort. The primary outcome measures were incidence of PVD, incidence of RT and incidence of RRD. Secondary aims were (i) to identify the incidence of late or secondary tears occurring after initial assessment (ii) to determine the incidence of tears not visible on slit-lamp biomicroscopy and requiring indirect ophthalmoscopy with scleral depression for detection and (iii) patient factors associated with retinal tear formation which might assist risk stratification and help design referral pathways.

## Methods

This was a prospective observational case series. Ethical approval was obtained from the East of England - Cambridge Central Research Ethics Committee and informed consent for participation in the study was obtained from all patients.

One thousand and ten patients with new symptoms of photopsia and floaters were recruited to the study over an eighteen month period. This included all patients referred from primary eye care services, optometrists, family doctors or hospital emergency services, in the catchment area of one district general hospital and one tertiary teaching hospital. Usual local practice is that all such patients attending hospital emergency services or family doctors would be referred either to hospital eye services or optometrists. For the purposes of this study, all local optometrists were invited to refer all patients with flashes and floaters for assessment in a dedicated research clinic. Patients were excluded if they were known to have a previous diagnosis of PVD or associated sequalae (retinal tear or detachment) in the symptomatic (index) eye.

Each patient completed a questionnaire regarding their ocular symptoms. A full and detailed ophthalmic history was taken, followed by a comprehensive ocular examination involving Snellen visual acuity assessment and auto-refraction, dilated slit-lamp biomicroscopy using SuperField NC Lens (Volk, Mentor, Ohio, USA) and then supine indirect ophthalmoscope fundus examination with scleral depression. A-scan measurements were recorded of the axial length of both eyes.

The diagnosis of PVD was made by the visualisation of the detached posterior hyaloid membrane (PHM) at slit-lamp biomicroscopy, with or without a condensing lens, with or without the presence of a complete or partial Weiss ring, according to published criteria [17, 18]. A definitive diagnosis was recorded for each patient. Retinal tears were defined as horseshoe shaped or operculated tears secondary to posterior vitreous detachment, and did not include atrophic round holes [19]. Those with no PVD were discharged. Those with PVD were reviewed two months later for a follow-up examination, (including indirect ophthalmoscopy with scleral depression in every case) whether or not the patient had new symptoms.

Data was collected and entered into an Excel spreadsheet (Microsoft Excel for Mac 2011) and analysed using StatPlus:Mac (Microsoft Excel for Mac 2011). The observations of the study are reported using descriptive statistics for the evaluation of the demographics, symptoms and clinical histories for each patient. Every patient was given a definitive diagnosis, from which sub-group evaluation was performed using the data from the affected eye of each patient. The sub-groups were:

1. PVD (only).
2. PVD with retinal tear.
3. PVD with retinal detachment.
4. Other (non-PVD) diagnoses.

For those situations where both eyes were affected, one eye was randomly chosen for the analysis. Sub-group statistical comparisons were performed using one-way analysis of variance (ANOVA) and statistical significance was set at a p-value of less than 0.05. Due to the unequal sample sizes for the four sub-groups, the Scheffe Test was chosen as the post-hoc method for determining which specific pairs of groups demonstrated the statistically significant difference as indicated by ANOVA.

## Results

In total, 1010 patients were recruited for the study. Of these, 613 patients (61%) were female and 397 (39%) were male. The median age was 64 years (range 20–94 years). The overwhelming majority of patients (95.6%) were Caucasian. The majority of patients (57%) were recruited from community optometrist practice, 24.9% from primary care physicians (General Practice), 12% from general hospital Emergency Departments (ED) and 6% were patient initiated self-referrals.

Symptoms were unilateral in 925 (92%) patients (479 (47%) in their left eye and 446 (44%) in their right eye) and bilateral in 85 (8%) patients. Both photopsias and floaters were reported by 622 (62%) patients, 315 (31%) reported floaters alone and 73 (7%) reported photopsias alone. Characteristics of the photopsias are summarised in eTable 1, and characteristics of the floaters summarised in eTable 2. The mean duration of symptoms was 52 days (standard deviation 121 days), with a median of 21 days. 723 (72%) patients noticed no deterioration in their vision; 266 (26%) reported some blurring and 12 (2%) reported definite loss of vision.

No previous eye problems or surgery were reported by 786 (77.8%) of the cohort. A history of blunt trauma was present in 24 (2.3%) patients, 10 (1%) reported a history of penetrating eye injury and 23 (2.3%) reported a history of intraocular inflammation. With regard to the fellow eye, 26 (2.6%) had a history of retinal detachment and 14 (1.4%) a history of retinal tear. A family history of retinal detachment or tear was present in 49 (4.9%) patients.

A diagnosis of symptomatic PVD was made in 896 (89%) patients at initial assessment, of which 807 did not have RT or RRD (80% of the total cohort, 90% of eyes with PVD). Symptomatic retinal tears were present in 89 patients (8.8% of total cohort, 9.9% of eyes with PVD), eight of whom had retinal detachment as a direct consequence (0.8% of total cohort, 0.9% of eyes with PVD). No abnormality was identified in 55 (5%) patients, and another 54 (5%) patients had other diagnoses including Fuch’s heterochromic iridocyclitis, uveitis, vitreous syneresis and one case of retinal dialysis.

Of the 896 patients with PVD, 132 (15%) also had ‘asymptomatic’ PVD in their fellow eye at initial assessment; i.e., a clinical finding of PVD in the fellow eye but no recall of prior symptoms on direct questioning. Of this subgroup of asymptomatic PVD, seven (5.3%) had retinal tears that required treatment (0.7% of total cohort), but there were no cases of retinal detachment. Of the eight eyes with retinal detachment, the fellow eye was healthy in four eyes, two had a history of detachment, one had a history of retinal tear and one had asymptomatic PVD. Of the 81 eyes diagnosed with retinal tear(s), three (3.7%) of the fellow eyes had asymptomatic retinal tears identified, and a further eight (10%) had asymptomatic PVD.

Slit-lamp biomicroscopy identified all patients with retinal detachment and 75 of 88 (85%) eyes with retinal tears (both symptomatic and asymptomatic). In 13 (15%) eyes, tears were not identified by slit-lamp biomicroscopy but were identified by indirect ophthalmoscope fundus examination with scleral depression. Indirect ophthalmoscope fundus examination with scleral depression identified all cases of RRD and 83 of 86 (97%) eyes with retinal tears (both symptomatic and asymptomatic). Three (3%) eyes with tears were not identified by indirect ophthalmoscope fundus examination with scleral depression but were identified by slit-lamp biomicroscopy. Two patients with retinal tears could not tolerate indented examination; and five (0.5%) of the total cohort could not tolerate indented examination.

Follow-up data was available for 729 of 901 (81%) patients asked to attend. Twenty-one patients developed retinal tears after their initial assessment representing a delayed retinal tear rate of 3% at two months. Six of the 21 (29%) eyes with delayed retinal tears also developed further retinal tears later. Nine patients who had retinal tears at initial assessment developed further retinal tears by final follow-up, representing a further retinal tear rate of 11% at two months. Nine new asymptomatic PVDs were diagnosed at follow-up, and new symptomatic PVDs were diagnosed in 21 eyes at follow-up. None of these eyes developed sequelae.

The presence of a detached posterior hyaloid membrane [18], Weiss ring and pigment [not including red blood cells] within the vitreous cavity was compared for each of the 1010 affected eyes within the study. Table 1 shows the relationship between these clinical signs and the diagnosis made.

**Table 1:** Posterior hyaloid membrane (PHM), Weiss ring (WR) and pigment in vitreous cavity for each diagnosis.

Diagnosis	*N* =	PHM	WR	Pigment
Retinal Dialysis	1	−	−	+
RRD	7	+	+	+
RRD	1	+	−	+
RT	55	+	+	+
RT	6	+	−	+
RT	19	+	−	−
RT	1	−	−	−
PVD (lattice, holes)	9	+	+	+
PVD (nil found)	2	+	+	+
PVD (nil found)	696	+	+	−
PVD (nil found)	80	+	−	−
PVD (nil found)	20	−	+	−
Impending PVD	5	−	−	−
Other (aphakic)	1	−	−	+
Other (nil found)	107	−	−	−

*RRD* Rhegmatogenous Retinal Detachment, *RT* Retinal Tear, *PVD* Posterior Vitreous Detachment.

The presence of pigment within the vitreous cavity (Shafer’s sign) was identified in all eight RRDs, the aphakic eye and the retinal dialysis. Shafer’s sign was present in 61 (75%) eyes with newly diagnosed retinal tears and also in 11 (1.3%) of eyes with PVD but no retinal tear or detachment. Nine of these 11 eyes demonstrated lattice generation or atrophic holes, but none developed retinal tear or detachment during follow-up. A Weiss ring was absent in 1 (12%) of the eyes with retinal detachment and 26 (32%) of the eyes with retinal tears.

Four diagnostic sub-groups were used for the purposes of statistical comparison: Retinal Detachment (RRD) (*n* = 8); Retinal Tear (RT) (*n* = 81); Posterior Vitreous Detachment (PVD) (*n* = 807); Other diagnoses (*n* = 113). Table 2 shows the difference in the demographics and the distribution of ocular symptoms between the four groups. Table 3 compares the past ocular history and Table 4 compares the ocular signs between the sub-groups.

**Table 2:** Diagnostic sub-group comparison of demographics and ocular symptoms.

	RRD (*n* = 8)	RT (*n* = 81)	PVD (*n* = 807)	Other diagnoses (*n* = 113)
Referral Source (%) (Optometrist: primary care physician/GP):ED:Self)	25: 38:12:25	64:16:15:5	55:26:12:6	62:24:8:6
Age (mean (SD) [range], in years)	59 (11.4) [44–76]	63 (7.5) [40–82]	64 (8.9) [32–94]	49 (15.1) [20–88]
Sex (male:female)	88:12	51:49	37:63	40:60
Age of needing reading glasses (mean (SD) [range], in years)	49.4 (6.7) [40–58]	48.8 (8.7) [9–64]	47.7 (11.5) [5–82]	41.3 (13.7) [5–60]
Affected eye (one:both)	89:11	96:4	94:6	73:27
Symptoms (%) (flashes:floaters:both)	0:63:37	4:44:52	6:28:66	16:43:41
Duration of flashes (%) (<1 second:seconds:minutes:constant)	0:33:0:67	44:29:20:7	48:37:9:6	34: 27:24:15
Flash morphology (%) (lightning:arc:stripe:other)	33:33:33:0	42:20:13:25	51:23:11:15	29:18:16:36
Precipitant of flashes (%) (spontaneous:head turn:eye movement:other)	0:33:0:67	47:9:24:20	42:14:23:20	53:8:15:24
Type of floaters (%) (spots: cobwebs: strands: other)	50:0:25:25	18:17:15:50	24:18:15:43	24:8:23:41
Duration of symptoms (mean (SD) [range] days)	32 (46) [1–120]	34 (49) [1–270]	46 (108) [1–1800]	112 (211) [1–1480]
Duration of symptoms (median days)	7	21	21	30
Nature of vision (%) (same:blur:missing)	24:63:13	54:42:4	74:24:2	68:29:3

*RRD* Rhegmatogenous Retinal Detachment, *RT* Retinal Tear, *PVD* Posterior Vitreous Detachment.

**Table 3:** Diagnostic sub-group comparison of ocular history.

	RRD (*n* = 8)	RT (*n* = 81)	PVD (*n* = 807)	Other diagnoses (*n* = 113)
Past ocular history (nil:blunt injury:penetrating:other) (%)	50:37:0:13	90:4:2:6	92:2:1:6	87:4:3:9
Cataract surgery (%)	38	6	9	6
Refractive laser (%)	13	7	3	3
Family history of RRD (%)	13	6	5	4

*RRD* Rhegmatogenous Retinal Detachment, *RT* Retinal Tear, *PVD* Posterior Vitreous Detachment.

**Table 4:** Diagnostic sub-group comparison of ocular signs.

	RRD (*n* = 8)	RT (*n* = 81)	PVD (*n* = 807)	Other diagnoses (*n* = 113)
Spherical Equivalent Refraction (Mean (SD) [Range) in Dioptres)	−2.5 (1.9) [−5.25 to 0]	−1.0 (2.7) [−8.75 to +4.00]	−0.8 (2.87) [−17.50 to +6.50]	−1.1 (3.60) [−9.00 to +21.50]
Axial length (Mean (SD) [Range] in mm)	25.1 (0.64) [24.0–25.7]	24.0 (1.4) [21.0–27.5]	23.7 (1.4) [20.9–30.5]	23.3 (2.8) [19.0–29.1]
AC depth (Mean (SD) [Range) in mm)	3.2 (0.40) [2.8–3.8]	3.1 (0.47) [2.3–4.6]	3.1 (0.49) [2.05–5.11]	3.1 (0.53) [2.29–4.68]
Pseudophakic (%)	25	6.2	8.9	3.7
Cataract (%) (nuclear sclerosis : blue-green : other : none)	25:38:10:25	45:21:11: 23	58:6:12:23	31:2:8:59

*RRD* Rhegmatogenous Retinal Detachment, *RT* Retinal Tear, *PVD* Posterior Vitreous Detachment.

Patients diagnosed with retinal detachment were predominantly male (*p* < 0.02) and reported blurred and missing vision more than the other groups (*p* < 0.001). Patients with retinal tears also reported a higher rate of blurred vision compared with the PVD group (*p* < 0.001). Patients without retinal detachment, retinal tear or PVD were significantly younger (*p* < 0.001), their symptoms were more likely to be bilateral in nature (*p* < 0.001) and they had a longer duration of symptoms, with a mean value of 112 days and a median value of 30 days (*p* < 0.001). The patients with PVD had higher reported combined photopsia and floaters than the ‘other diagnoses’ group. No other statistically significant differences were identified.

Patients with retinal detachment were more likely to have had cataract surgery (*p* < 0.05), but differences in history of ocular injury, refractive laser or family history of retinal detachment did not meet statistical significance.

**Fig. 1:**
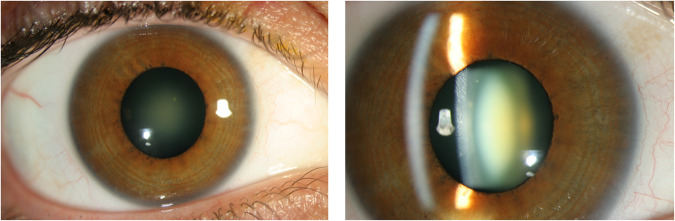
Blue-green nuclear cataract. Photograph at slit-lamp demonstrating blue-green nuclear cataract which, when present in middle-aged myopes, indicates a higher risk of retinal tear when posterior vitreous detachment occurs [20].

Eyes with retinal detachment had significantly longer axial lengths (*p* < 0.05) but the degree of myopic refractive error was not statistically significant. Eyes presenting with retinal detachment and retinal tear were significantly associated with a higher rate of blue-green nuclear cataract [20] (Fig. 1) compared to those eyes with purely a PVD or ‘other diagnoses’ (*p* < 0.001). Eyes with ‘other diagnoses’ were less likely to have cataract of any type (*p* < 0.001).

## Discussion

This study reports the result of what is to our knowledge the largest prospective case series analysis of acute PVD in a community setting aiming to reflect a representative cohort of patients with acute PVD in a general population. The study was specifically designed to provide PVD data from as broad as possible a range of community presentations and mitigate the potential bias associated with previous studies based in specialist tertiary referral centres, as referrals from all community sources including optometrists, family doctors as well as hospital emergency departments, were pooled into the PVD clinic without filtering through other ophthalmologists. It was conducted prospectively to mitigate some of the data quality problems associated with retrospective analysis. Although every effort was made to recruit all patients prospectively to the study cohort, including and especially those who would not normally be referred, it is likely some patients will have declined or been unable to attend for assessment and so the denominator for the entire study population is unknown.

A single clinical examiner will have reduced inter-observer variability and combining slit-lamp biomicroscopy with indirect ophthalmoscopy and scleral depression for every case provides collateral insurance that retinal tears (where present) will have been identified. The population of the region studied is 95% Caucasian, so there is uncertain applicability to other ethnicities where the incidence of retinal detachment is known to be different [21–24]. There was a 19% loss to follow-up in the patients diagnosed with PVD, and although any with acute retinal detachment would be expected to re-present promptly and quickly on the basis of advice given to all patients at their initial attendance and examination, the clinical outcome for this group is otherwise unknown. The mean and median symptom duration of 52 and 21 days are relevant to note, in that patients who progress rapidly to retinal detachment are likely to have presented acutely with symptomatic retinal detachment, and would thus not be included in this study, suggesting that the true incidence of retinal tears in posterior vitreous detachment may be higher than that indicated here.

We found a retinal tear rate of 8.8% of patients with photopsia and floaters and in 9.9% of all PVDs. Other studies have found rates of PVD-related retinal tears between 6.4% and 46% [5, 7, 9, 10, 12–16]. The results of the current study are at the lower end of this range and are likely to reflect a representative estimate from a large general community population rather than study cohorts drawn from specialist tertiary referral vitreoretinal centres.

Delayed retinal tears, present at two-months follow-up but not at initial presentation, occurred in 3% of our cohort. This is comparable to figures from other studies showing a delayed retinal tear rate of 0–5.9% [10–14, 16]. As this is a relatively low rate, it may not be necessary to routinely follow-up uncomplicated posterior vitreous detachments in the absence of new symptoms or significant risk factors in the patient’s history, examination or family history. Importantly, of the patients with delayed retinal tears, a third (29%) went on to develop further retinal tears. This may suggest that the PVD is progressing more slowly but pathologically in these patients and represent a key sub-group that require longer follow-up than the main cohort.

In patients with PVD, 15% had asymptomatic PVD in the fellow eye, of whom 5% had associated retinal tears. This figure is similar to that previously reported by Hikichi who found 20% of fellow eyes had a PVD with retinal tears in 4% [7]. Boldrey et al. found a higher fellow eye retinal tear rate of 2.6% [8], but the patients were referred from a retinal practice and the overall retinal tear rate was 18.5% which may represent patient selection bias. All these studies, including the results of the present study show the importance of dilated fundal examination of both eyes in patients presenting with symptomatic PVD in one eye.

A further key finding is that one third (32%) of eyes with retinal tears had no identifiable Weiss ring as a feature of their PVD, the ring presumably being destroyed during the process of separation of the PHM from the surface of the retina. This is further evidence supporting numerous previous studies that the absence of a Weiss ring cannot be taken as a reliable and necessary indication of PVD [17, 18, 25, 26]. The fact that 85% of tears were seen with slit-lamp bio-microscopy and 97% with indirect ophthalmoscopy with scleral depression, which was tolerated in 99.5% of patients, reinforces that both methods are complimentary to identify all retinal breaks. This is comparable with a smaller study which found 89% detection rate with slit-lamp biomicroscopy [27].

In keeping with other studies [12, 15, 16], being male, having blurred vision, having long-axial length, and pseudophakia increased the risk of finding retinal tears or detachment in association with PVD. However, this study also highlights the significant association with the presence of blue-green nuclear cataract (Fig. 1), characteristically present in middle-aged myopes [20], who should be warned of their high risk of retinal tear when they develop symptoms of PVD.

This large, prospective community-based study shows that patients have a 9.9% chance of associated retinal tear and/or detachment at the time of PVD and in 15% of these cases, the tears were only visible using indirect ophthalmoscopy and scleral depression. A further 3% of patients developed retinal tears in the two month period after the initial assessment. This study provides important data on which to base assessment of PVD in a non-tertiary referral community setting.

## Summary

### What was known before

- Retinal tear secondary to posterior vitreous detachment is a treatable cause of rhegmatogenous retinal detachment.

### What this study adds

- Symptomatic posterior vitreous detachment has a 9.9% risk of retinal tear, of whom 11% will develop further tears. 3% of patient with no tears initially will develop tears by two months of subsequent follow-up.
- Weiss ring is absent in one-third of patients with retinal tears, but visible posterior hyaloid membrane is diagnostic for posterior vitreous detachment.
- Presence of blue-green nuclear cataract is a significant risk factor for developing retinal tears after posterior vitreous detachment.
- 15% of retinal tears were only found on indented indirect ophthalmoscope examination, so this is a critical part of the examination, along with examination of the fellow eye.

### Supplementary information

Supplemental Table 1

Supplemental Table 2

## Data Availability

The datasets generated during and/or analysed during the current study are available from the corresponding author on reasonable request.
