# Supplementary material for: Posterior vitreous detachment and retinal tear – a prospective study of community referrals
Source: Eye (Lond). 2023 Oct 5;38(4):786–91. doi: 10.1038/s41433-023-02779-3 (PMC10920725; doi:10.1038/s41433-023-02779-3)
Supplement: Supplementary file 1 — Supplemental Table 1 [file 41433_2023_2779_MOESM1_ESM.docx]

**Supplementary Table 1: Flashes - specific characteristic features (n=695 patients).**

| Frequency | 63% daily | 25% one-off | 9% weekly | 3% monthly |
| --- | --- | --- | --- | --- |
| Duration | 46% <1second | 35% seconds | 12% minutes | 7% constant |
| Morphology | 48% lightning streak | 23% arc | 12% stripe | 17% variety of other shapes |
| Location | 83% temporal | 10% central | 3% entire field | 4% other locations |
| Onset | 43% spontaneous | 23% eye movement | 13% head turn | 21% combination or not applicable |
| Most noticeable | 51% in dark | 28% light and dark | 17% in light | 4% neither |
| Colour | 63% white | 15% yellow | 8% gold | 14% other colours |
