# Supplementary material for: Posterior vitreous detachment and retinal tear – a prospective study of community referrals
Source: Eye (Lond). 2023 Oct 5;38(4):786–91. doi: 10.1038/s41433-023-02779-3 (PMC10920725; doi:10.1038/s41433-023-02779-3)
Supplement: Supplementary file 2 — Supplemental Table 2 [file 41433_2023_2779_MOESM2_ESM.docx]

**Supplementary Table 2: Floaters - specific characteristic features (n=937 patients).**

| Number | 63% between 1 and 10 | 26% solitary floater | 9% hundreds to thousands | 2% between 10 and 100 |
| --- | --- | --- | --- | --- |
| Type | 23% spots or dots | 17% cobwebs | 16% strands | 30% combination  14% others |
| Colour | 69% black | 10% opaque | 7% brown | 14% other colours |
